# Empathy in Undergraduate Medical Students of Bangladesh: Psychometric Analysis and Differences by Gender, Academic Year, and Specialty Preferences

**DOI:** 10.1155/2014/375439

**Published:** 2014-04-07

**Authors:** Asma Mostafa, Rozina Hoque, Mohammad Mostafa, Md. Mashud Rana, Faisal Mostafa

**Affiliations:** ^1^Department of Anatomy, Chattagram Maa-O-Shishu Hospital Medical College, Chittagong, Bangladesh; ^2^Department of Pharmacology & Therapeutics, Chattagram Maa-O-Shishu Hospital Medical College, Chittagong, Bangladesh; ^3^Department of Psychiatry, Chattagram Maa-O-Shishu Hospital Medical College, Chittagong, Bangladesh; ^4^Department of Pharmacology & Therapeutics, Chittagong Medical College, Chittagong, Bangladesh; ^5^Department of Surgery, Diabetic Association Medical College, Faridpur, Bangladesh

## Abstract

Empathy is considered to be associated with better patient compliance, satisfaction, and clinical outcomes. The aim of the study is to measure and examine empathy among a sample of undergraduate medical students of Bangladesh. It was a cross-sectional study and all the medical students of first through fifth year enrolled at Chattagram Maa-O-Shishu Hospital Medical College during the study period of 2014 were surveyed. Participants anonymously completed the Jefferson Scale of Empathy Medical Student version translated into Bengali language, a valid and reliable 20-item self-administered questionnaire. Principal component factor analysis with varimax rotation and Cronbach's alpha coefficient were calculated to check validity and reliability of the scale. ANOVA was used to examine the differences in empathy between gender, academic years, and specialty preferences. The mean empathy score was 110.41 ± 13.59. Cronbach's alpha coefficient was 0.88. There were significant associations between gender and empathy scores. The level of empathy in medical students gradually increases after clinical training in medical college. A nonsignificant difference was found between empathy scores and specialty preferences. It is suggested that the medical curriculum in Bangladesh should include more extensive program to promote empathy and other humanistic values among the medical students.

## 1. Introduction

Empathy is the ability to understand and share the feelings of others [1]. It is the power of entering into others' personality and imaginatively experiencing their emotional state. Hojat et al. define empathy as “*a predominantly cognitive attribute that involves understanding of the patient's experiences, concerns, and perspectives, and a capability to communicate this understanding. An intention to help by preventing and alleviating pain and suffering is an additional feature of empathy in the context of patient care*” [2]. Empathy is essential in “physician-patient relationship” to produce a desire within physician to help the patient, to know what the patient is thinking or feeling, to provide best care to the patient, and to blur the line between physician and patient [3]. Empathy is important in development of interpersonal understanding which allows the patient to feel respected and validated [3]. It is the key element which can propel the physician toward altruistic action [4]. It helps the physician to be closer to patient, putting the benefit of other above those of self, even at some sacrifice to one self. Empathy is considered to be associated with improved health outcomes. A good physician-patient relationship is capable of creating better patient compliance, satisfaction, and clinical outcomes [5].

There are various factors determining empathy, such as, age, gender, family background, culture, intelligence, emotional stability, and education [6, 7]. The study of empathy in patient care is important not only within a society but also among different cultures because of variations in medical education curriculum, cultural norms, and social learning.

One of the major tasks in medical education is to maintain and increase empathy in medical students for patients. But various researches suggest that empathy in medical students decreases during the course of medical training [8–10]. Students experience medical training as stressful which might be injuring instead of fostering empathy. [11]. During this training, students also learn how to manage the stresses and anxiety of illness which may develop maladaptive responses that lead to a decline in their level of empathy [12]. Besides this, dependence on technology for diagnosis and limited interactions with patients may lead to a decrease in empathy among the medical students [13].

Empathy is believed to be measurable and teachable. The Association of American Medical Colleges recommended that empathy should be integrated and assessed in medical education [2, 14]. Various research instruments, for example, Interpersonal Reactivity Index, Balanced Emotional Empathy Scale, and Jefferson Scale of Empathy (JSE), are available to measure empathy [11]. Among the self-reported instruments, JSE was used by various researchers to measure empathy specifically within the context of the physician-patient relationship [7, 15–20]. The validity and reliability of the JSE have been also reported [18, 21].

Exercises and a more extensive program have been introduced formally in various medical curricula worldwide, to asses and promote empathy and other humanistic values among the medical students [12]. Such programs are still not well established in Bangladesh. The present study was designed to examine and measure empathy among a sample of undergraduate medical students of Bangladesh and also to compare the effects of gender, academic year, and specialty preferences on empathy.

## 2. Method

The study was cross-sectional in nature and carried out in Chattagram Maa-O-Shishu Hospital Medical College (CMOSHMC), Chittagong, Bangladesh, during the study period of 2014. The study was approved by the CMOSHMC Ethical Committee.

### 2.1. Participants

All medical students of first through fifth year enrolled at CMOSHMC during 2014 were eligible to participate in the study. Participants included 426 medical students from five academic years, in the first (*N* = 100), second (*N* = 88), third (*N* = 86), fourth (*N* = 77), and fifth year (*N* = 75). There were 291 female and 135 male students in the study population. The undergraduate medical curriculum in Bangladesh is a traditional five year medical school training with 1.5 years preclinical study, 2 years paraclinical study with limited patient contact, and 1.5 years clinical study.

### 2.2. Instruments

The JSE has been adapted to several countries and languages [7, 15–18, 20]. It exists in three versions, HP-Version: for administration to physicians, S-Version: for administration to medical students, HPS-Version: for administration to students in other health professions like nursing, paramedical courses [2]. In this study, the Jefferson Scale of Empathy Medical Student version (JSE S-version) translated into Bengali language was used to measure medical students' attitudes toward empathic physician-patient engagement in the contact of patient care. The JSE S-version was translated into Bengali language using a back translation procedure to ensure the accuracy of the translation [2, 7, 15–20, 22, 23]. At first, JSE S-version was translated from English into Bengali language by two bilingual researchers having a detailed understanding of the instrument. Later the translated Bengali version was sent to another three bilingual researchers who had not seen the original English version and were asked to translate Bengali version back into English. Then the original English version was compared with the back-translated English versions to see consistency and adaptations that were done in wording, where needed, to make the text consistent with Bengali culture without losing intended key concepts, and lastly a final Bengali version was produced. The JSE S-version includes 20 items each answered on a seven-point Likert scale. 10 positively worded items were linked to “perspective taking” and directly scored (1 = strongly disagree, 7 = strongly agree). 10 items were negatively worded and reversed scored (1 = strongly agree, 7 = strongly disagree) [13]. Eight of the ten negatively worded items were concerned with “compassionate care” and 2 items were linked to “standing in the patient's shoes.” Scores ranged from 20 to 140. Higher values indicate a higher degree of empathy [11].

Specialties were categorized into three groups like people-oriented, technology-oriented, and other specialties, as shown in Table 1 [2, 10, 23]. Students specified their career specialty intentions, in terms of possibility of entering each of the specialties.

### 2.3. Procedures

Before going into the survey, it was explained to the participants that the questionnaire was about empathy asking for their personal beliefs and opinions and that the results would be used for research purpose. The participants were also informed that the study would be anonymous, so it would not be possible to identify them anywhere in the questionnaire. Therefore, there was no need to answer any questions in a manner that would be viewed as a “good behavior” by others. It was also stated that participation was voluntary but returning the questionnaire would be taken as consent to participate. There would be neither reward for participating nor any penalty for not participating in the survey. Respondents did not need to sign their names, but they were asked to provide information on their gender and academic year. JSE S-version in Bengali questionnaire was distributed to the 1st, 2nd, 3rd, 4th, and 5th year medical students during their regular classes. It has been suggested that printed copies command greater response rates over electronic versions [24]. Participants were given 1 day to complete the study and also asked to take the test individually.

### 2.4. Statistical Analysis

Questionnaires missing information on five or more items were considered incomplete and excluded from the data for subsequent analysis [2]. If a respondent fails to answer 4 or fewer items, the missing values were replaced with the mean score calculated from the items the respondent completed [2]. After data collection, frequency distributions, central tendencies, and dispersions of the Bengali version of JSE S-version scores were determined. Cronbach's alpha coefficient was calculated to assess the internal consistency aspect of reliability of the instrument. A reliability of 0.70 and higher was considered satisfactory [7, 14, 20, 25]. Correlation between each item and the total score (item-score correlation) was calculated. Principal component factor analysis with varimax rotation was used to search for the underlying factor structure of the Bengali version of the JSE S-version. Eigenvalues greater than 1 were required to retain factors [26] and factor loadings of 0.35 or greater were required for the interpretation of the factor structure [14]. ANOVA with post hoc test analysis was used to examine the differences of empathy scores related to gender, academic years, and specialty preferences. Data were analyzed using SPSS version 11.5 and a *P* value <0.05 was set as statistically significant.

## 3. Results

348 questionnaires were returned out of possible 426. The overall response rate was 81.69%.  Table 2 shows that first year students have the highest response rate and fifth year students have the lowest response rate.

Range, mean ± SD, and quartile points of the Bengali version of JSE S-version empathy scores are shown in Table 3. Cronbach's alpha coefficient is in an acceptable range which indicates that Bengali version of JSE S-version was internally consistent for psychological measures.

The Kaiser-Meyer-Olkin (KMO) analysis was performed, yielding an index of 0.89. The result for Bartlett's test of sphericity was 2036.40 and was highly significant (*P* = 0). This information indicates the appropriateness of principal components analysis. Summary results of principal component factor extraction with varimax rotation of data for the 20 items of the Bengali version of JSE S-version are shown in Table 4. Three factors with eigenvalue >1 emerged and accounted for a total of 44.80% of the variance. Factor 1 loaded seven items having a factor coefficient of greater than 0.35, five items related to “perspective taking” and two items related to “compassionate care.” Factor 2 loaded eight items with a factor coefficient of greater than 0.35 for statements reverse-scored, six items related to “compassionate care” and two items related to “standing in the patient's shoes.” Factor 3 loaded with five items with a factor coefficient of greater than 0.35 related to “perspective taking.”

Table 4 also shows the mean ± SD, communalities (h2), and corrected item-total correlations for each item in the Bengali version of JSE S-version. The mean item score responses ranged from 4.14 to 6.39. The lowest score was observed for item 18 (reverse score) and the highest score was for item 2. These indicate that the students' responses tend to be skewed towards the upper end of the scale. The interitem score correlation was positive. Corrected item-total score correlation ranged from a low of 0.334 for item 8 (reverse score) to a high of 0.657 for item 18 (reverse score).

**Figure 1:**
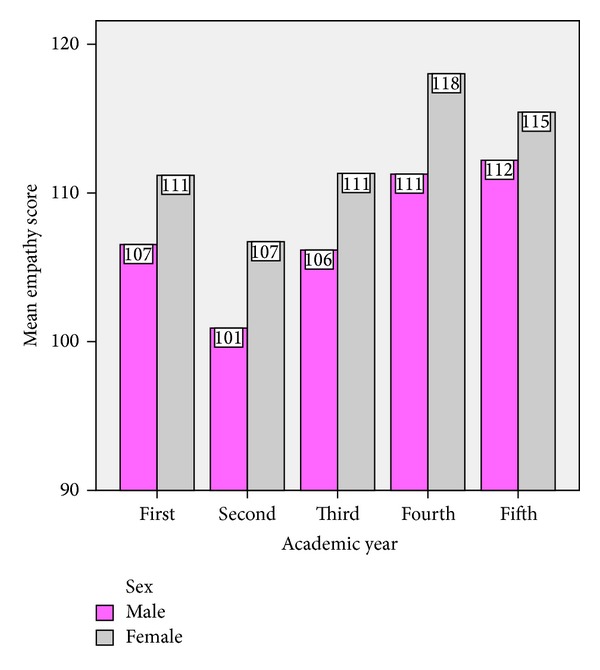
Bar diagram showing mean empathy score of medical students in each academic year by gender (*N* = 348).

Table 5 shows a statistically highly significant difference in mean empathy scores between female and male medical students.  Figure 1 shows that female medical students have higher empathy scores than male medical students of five academic years. ANOVA shows that gender had no effect on empathy score for medical students in first, third, and fifth years with *P* values 0.17, 0.11, and 0.40, respectively, but revealed a significant difference in fourth year medical students (*P* = 0.01) and nearly significant difference in second year medical students (*P* = 0.07).

**Figure 2:**
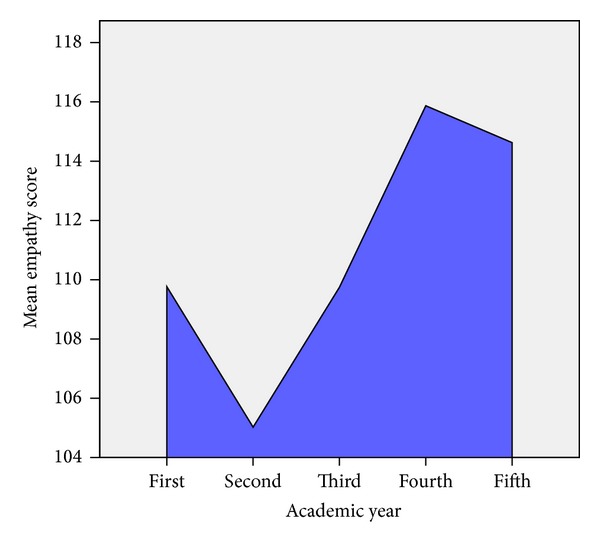
Graph showing mean empathy score of medical students in first to fifth academic years (*N* = 348).

Analysis of variance showed that there was a significant difference between mean scores from various academic years (Table 5). In addition, post hoc analysis showed a significant difference between second year and first (*P* = 0.02), third (*P* = 0.04), fourth (*P* = 0), and fifth years (*P* = 0). There was also a significant difference between third year and fourth year (*P* = 0.01). No difference is seen between fourth and fifth year students (*P* = 0.63).  Figure 2 shows that there is a decline in mean empathy scores from first to second year, followed by a gradual increase from third to fourth year. Although there is a decrease of mean empathy scores from fourth to fifth year, it was statistically nonsignificant.

A nonsignificant difference was found between medical students preferring “people-oriented” or “technology-oriented” or “other” specialties.

## 4. Discussion

The aim of this study was to examine levels of patient empathy in undergraduate medical students of Bangladesh. In Bangladesh there is no scale to examine empathy in medical students. In the present study, Bengali version of JSE S-version was adapted for Bangladeshi population. The reliability of Bengali version of JSE S-version was examined. Cronbach's alpha coefficient in this study (*r* = 0.88) was similar to those reported for Korean (*r* = 0.84) [16], Japanese (*r* = 0.80) [15], Chinese (*r* = 0.83) [22], and South African medical students (*r* = 0.79) [13]. This result indicates that Bengali version of JSE S-version is internally consistent in undergraduate medical students of Bangladesh. The principal component analysis showed a three-factor solution that was somewhat similar to the pattern in other studies. This also provided support for the construct validity of the Bengali version of JSE S-version.

The mean empathy score of this study (mean = 110.41) was similar to Chinese (mean = 109.60) [22] and higher than Japanese (mean = 104.3) [15], Kuwaiti (mean = 104.6) [27], and Iranian (mean = 105.1) [17] medical students. But the mean score of this study was lower than that of American medical students (mean = 115) [8]. It may be due to differences in cultural factors, custom, ethnicity, spiritual belief, educational system, variation of selection of medical students, and availability of appropriate role model.

The gender ratio of entire medical students at CMOSHMC is 68.30% female to 31.69% male. This gender ratio will be found in most of medical college in Bangladesh. Previous studies reported that female medical students are more empathic than their male counterparts [6, 10, 13, 15, 16, 22, 28]. Finding of this study is also consistent with most of the other studies. The gender difference is due to evolutionary-biological gender characteristics, styles in interpersonal care, socialization, and gender role expectations [13]. Women show a greater understanding of the emotional support which is important to develop interpersonal relationships with patients.

There was statistically significant difference in empathy scores in different academic years with second year students having the lowest empathy scores. The first year and third year students had statistically significant higher empathy scores than those of second year. This indicates that level of empathy declines in preclinical year followed by a rise of empathy scores in paraclinical year when the students experienced first limited contact with patients. The fourth year had the highest empathy scores followed by a nonsignificant decrease in the fifth year. Although the findings of this study regarding enhancement of empathy are not in agreement with American [8, 10] medical students, they are similar to those of Chinese [22], Japanese [15], Korean [16], Portuguese [11], and Kuwaiti [27] medical students. A joint family culture is still predominant in Bangladesh. The age range of the participants of this study is 19 to 24 years. In Bangladesh, person of this age range is usually part of a joint family. They have optimal family support for their living arrangements. So, they can continue their education with less stress and exhaustion. In a study, Kataoka et al. [23] reported that physicians living in joint family are more empathic than those living in a nuclear family. Morling and Lamoreaux [27] have reported that Asians have more “collectivistic and less individualistic social cultures” than Westerners. This may be the reason that empathy scores of the medical students of this study gradually increases after clinical training.

In this study, there was a nonsignificant difference between specialty preferences and empathy scores. However, those students who choose “people-oriented” and “technology-oriented” specialty had slightly higher mean empathy scores than those who preferred “other specialty.” Such observations are also reported in Kuwaiti medical students [27]. Students who find themselves to be less comfortable with patients usually choose “technology-oriented” or “other specialty.”

This study was cross-sectional in nature. Here empathy level is examined in five different academic years. A prospective study is needed to follow students annually from the beginning of first year until graduation, to find out an accurate image of change in empathy levels. The present study only focuses on students attending a private medical college in Bangladesh. Larger study populations covering different medical colleges of Bangladesh are needed to validate the results of this study.

## 5. Conclusion

The results of this study provide support for the reliability and construct validity of the Bengali version of JSE S-version. There were significant associations between gender and empathy scores. The level of empathy in medical students gradually increases after clinical training in medical college. It is suggested that the medical curriculum in Bangladesh should include more exercises and extensive program to promote empathy and other humanistic values among the medical students.

## Figures and Tables

**Table 1:** Career preference categories.

People-oriented specialties	Technology-oriented specialties	Other specialties
Internal medicine and medicine subspecialties: cardiology, neuromedicine, nephrology, endocrinology, and gastroenterology	General surgery and surgical subspecialties: ophthalmology, orthopaedics surgery, urology, neurosurgery, plastic surgery, ENT, and pediatric surgery	Pharmacology/forensic medicine/community medicine/anatomy/physiology
Psychiatry/dermatology	Anesthesiology/radiology/physical medicine/oncology	Undecided
Pediatrics	Obstetrics and gynecology	
General practitioner	Pathology/microbiology/hematology/biochemistry	

**Table 2:** Socio-demographic characteristics of the participants.

Characteristics	Number of students	Number of responders	Response rate (%)
Academic Year			
1st	100	98	98
2nd	88	82	93.18
3rd	86	59	68.60
4th	77	69	89.61
5th	75	40	53.33
Gender			
Male	135	104	77.04
Female	291	244	83.85

**Table 3:** Descriptive statistics for the Bengali version of the JSPE-S.

Statistics	Value
Range	69.00–135.00
Mean ± SD	110.41 ± 13.59
25th percentile	100.00
50th percentile	114.00
75th percentile	121.00
Reliability (Cronbach's alpha coefficient)	0.88

*N* = 348.

**Table 4:** Principal component analysis with varimax rotation and corrected item-total correlations of the Bengali version of JSE S-version.

Items	*F*1	*F*2	*F*3	Mean ± SD	Communalities *h*2	Corrected item-total correlations *r*_i-t_
2	0.720	0	0	6.39 ± 0.75	0.572	0.556
1R*	0.718	0	0	5.04 ± 1.76	0.656	0.649
20	0.686	0	0	6.17 ± 0.89	0.591	0.562
19R	0.671	0	0	5.89 ± 1.40	0.476	0.418
4	0.424	0	0	6.01 ± 0.87	0.297	0.456
10	0.423	0	0	6.06 ± 0.88	0.262	0.422
13	0.382	0	0	5.82 ± 0.94	0.206	0.361
6R	0	0.721	0	4.25 ± 1.48	0.635	0.646
18R	0	0.694	0	4.14 ± 1.76	0.612	0.657
3R	0	0.658	0	4.72 ± 1.33	0.442	0.428
14R	0	0.652	0	4.93 ± 1.66	0.481	0.528
7R	0	0.528	0	5.46 ± 1.42	0.430	0.561
8R	0	0.520	0	5.00 ± 1.36	0.325	0.334
12R	0	0.494	0	5.68 ± 1.16	0.297	0.421
11R	0	0.407	0	6.06 ± 1.16	0.360	0.533
16	0	0	0.698	5.68 ± 0.90	0.531	0.386
17	0	0	0.638	5.47 ± 1.16	0.474	0.436
9	0	0	0.560	5.83 ± 1.13	0.495	0.508
15	0	0	0.553	5.83 ± 1.07	0.369	0.408
5	0	0	0.487	5.98 ± 1.02	0.449	0.437

% of variance	31.29	7.46	6.05			
Alpha	0.75	0.81	0.68			

*R: items were reverse scored.

The factor pattern coefficients of 0.35 and below were replaced by zeros.

**Table 5:** Group differences of the Bengali version of JSE S-version.

Group	*N*	Range	Mean ± SD	Statistical difference	
				*F* value	*P* value^†^
Gender					
Male	104	72.00–135.00	106.72 ± 14.33	11.28	0.00 (S)^††^
Female	244	69.00–134.00	111.99 ± 12.97		
Academic Year					
1st	98	75.00–132.00	109.77 ± 15.29	7.592	0.00 (S)
2nd	82	69.00–133.00	105.02 ± 14.37		
3rd	59	85.00–129.00	109.75 ± 11.39		
4th	69	92.00–134.00	115.87 ± 10.72		
5th	40	90.00–135.00	114.63 ± 10.30		
Specialty preferences					
People-oriented	146	69.00–133.00	110.45 ± 13.87	0.07	0.93 (NS)*
Technology-oriented	119	72.00–135.00	110.68 ± 13.28		
Other	83	75.00–133.00	109.96 ± 13.69		

^†^Significance of correlation was tested at 5% level (*P* = 0.05).

^††^S: significant; *NS: nonsignificant.

## Abbreviations

JSE: Jefferson Scale of Empathy
CMOSHMC: Chattagram Maa-O-Shishu Hospital Medical College
JSE S-version: Jefferson Scale of Empathy Medical Student version.
